# Diagnostic anticipation to reduce emergency department length of stay: a retrospective cohort study in Ferrara University hospital, Italy

**DOI:** 10.1186/s12913-020-05472-3

**Published:** 2020-07-08

**Authors:** Andrea Strada, Niccolò Bolognesi, Lamberto Manzoli, Giorgia Valpiani, Chiara Morotti, Francesca Bravi, Roberto Bentivegna, Elena Forini, Antonella Pesci, Armando Stefanati, Eugenio Di Ruscio, Tiziano Carradori

**Affiliations:** 1 Emergency Department, S. Anna University Hospital of Ferrara, Ferrara, Italy; 2 School of Hygiene and Preventive Medicine, University of Ferrara, Ferrara, Italy; 3 Department of Medical Sciences, University of Ferrara, Ferrara, Italy; 4 Research Innovation Quality and Accreditation Unit, S. Anna University Hospital of Ferrara, Viale Aldo Moro 8, 44124 Ferrara, Italy; 5 Risk manager, S, Anna University Hospital of Ferrara, Ferrara, Italy; 6 Management Control, S, Anna University Hospital of Ferrara, Ferrara, Italy; 7 Hospital Direction, S, Anna University Hospital of Ferrara, Ferrara, Italy

**Keywords:** Emergency department, Overcrowding, Healthcare services research, retrospective cohort study, Quality improvement

## Abstract

**Background:**

Emergency Department (ED) crowding reduces staff satisfaction and healthcare quality and safety, which in turn increase costs. Despite a number of proposed solutions, ED length of stay (LOS) - a main cause of overcrowding - remains a major issue worldwide.

This retrospective cohort study was aimed at evaluating the effectiveness on ED LOS of a procedure called “Diagnostic Anticipation” (DA), which consisted in anticipating the ordering of blood tests by nurses, at triage, following a diagnostic algorithm approved by physicians.

**Methods:**

In the second half of 2019, the ED of the University Hospital of Ferrara, Italy, adopted the DA protocol on alternate weeks for all patients with chest pain, abdominal pain, and non-traumatic bleeding. A retrospective cohort study on DA impact was conducted. Using ED electronic data, LOS independent predictors (age, sex, NEDOCS and Priority Color Code, imaging tests, specialistic consultations, hospital admission) were evaluated through multiple regression.

**Results:**

During the weeks when DA was adopted, as compared to control weeks, the mean LOS was shorter by 18.2 min for chest pain, but longer by 15.7 min for abdominal pain, and 33.3 for non-traumatic bleeding. At multivariate analysis, adjusting for age, gender, triage priority, specialist consultations, imaging test, hospitalization and ED crowding, the difference in visit time was significant for chest pain only (*p* < 0.001).

**Conclusions:**

The impact of DA varied by patients’ condition, being significant for chest pain only. Further research is needed before the implementation, estimating the potential proportion of inappropriate blood tests and ED crowding status.

## Background

The American College of Emergency Physicians defines crowding as a need for emergency services exceeding available resources for patient care in the Emergency Department (ED), hospital or both [1]. In particular, ED crowding is considered a public health issue worldwide [2], because its consequences include diminished patients and staff satisfaction, decreased patients safety (delays in the evaluation and treatment of emergency patients, increased morbidity and mortality), increased costs, and reputation damage [1].

The causes of crowding are multifactorial and include, among the major contributors, the length of stay (LOS) of ED patients [3]. Evidence suggests that lengthy visits impact is more relevant than non-urgent [4] or frequent visits [5]. One of the main causes of prolonged ED LOS involves the patients flow within the ED and is defined as “throughput” [6]. This period starts from patient’s arrival in ED (triage) to the patient’s leaving of the ED.

Many interventions have been tested to improve ED waiting times and LOS [7], including deployment of physicians at triage [8], use of trained scribes to assist ED physicians [9], nurse-initiated diagnostic ordering at triage, based on physician approved algorithms [10], and resident-initiated advanced triage [11]. A systematic review concluded that nurse-initiated diagnostic ordering were effective in reducing ED LOS, but the available evidence was limited, as studies were scarce and of poor methodological quality [12].

Given that the Italian and Regional healthcare government recommended a maximum threshold of 6 hrs for ED LOS, the Ferrara University Hospital introduced nurse-initiated diagnostic ordering at triage at alternate weeks, thus allowing an evaluation of the impact and feasibility.

This retrospective cohort study was aimed at evaluating the impact on ED LOS of a procedure called “Diagnostic Anticipation” (DA), which consisted in anticipating the ordering of blood tests by nurses, at triage, following a diagnostic algorithm approved by physicians.

## Methods

### Ethics

The study protocol was approved by the Independent Ethical Committee of Area Vasta Emilia Centrale (CE-AVEC, study code: 840/2019/Oss/AOUFe; date of approval CE: 11/12/2019) and the study had an administrative permission by General Direction of Ferrara University Hospital.

### Study design and setting

This retrospective cohort study was performed at the ED of the Ferrara University Hospital, a tertiary care hospital in Emilia-Romagna Region, Northern Italy, from July 1st, 2019 to December 31, 2019. All participants were monitored during the ED stay, from triage registration to physician’s decision (hospital admission or discharge).

### Study population

Inclusion criteria were hour of visit between 8:00 am to 8:00 pm, a presenting complaint of chest pain, abdominal pain or non-traumatic bleeding and a triage priority color code yellow or green. In Italy, triage involves assigning a priority color code to patients arriving at the hospital ED: white (the situation is not an emergency, the patient is safe or does not have a life-threatening condition); green (the situation is not an emergency, the patient has an acute but stable pathology, and vital signs are normal); yellow (the situation is a medical emergency, intervention cannot be delayed); red (the situation is an absolute emergency, the patient’s vital signs have deteriorated or indicate an immediate threat to patient’s life) [13].

Exclusion criteria were an age < 18 years and patient’s death or leave of ED before physician’s decision.

### Procedure

The DA protocol was implemented on alternate weeks and between 8:00 am to 8:00 pm to evaluate its impact before full implementation (to avoid the history bias that typically afflicts before/after evaluations). During the weeks in which the DA was adopted, following an algorithm made by the physicians, the nurses at triage ordered the blood tests listed in Table 1 using a pre-determined command in the hospital software (SAP) for all eligible patients, before physician’s visit.

**Table 1:** Nurse-initiated blood test ordering at triage, based on a physician-approved diagnostic algorithm

Condition at triage	Blood tests
Chest pain	Complete blood count, creatinine, sodium, potassium, glycemia, cardiac troponin I
Abdominal pain	Complete blood count, creatinine, sodium, potassium, glycemia, Alanine Transaminases (ALT), bilirubin, C-Reactive Protein (CRP), pancreatic lipases
Non-traumatic bleeding	Complete blood count, creatinine, sodium, potassium, Prothrombin Time (PT), Partial Thromboplastin Time (PTT)

A multidisciplinary team including the hospital risk manager, ED physicians and nurses, laboratory physicians and IT technicians defined the standard operating procedure (additional File 1): whenever an eligible patient was accepted to ED triage, the nurse selected the above listed blood tests within 15 min. When blood tests results become available, the nurse delivered them to the physician for interpretation.

The blood tests and the presenting complaints were selected by the panel of experts analyzing the ED data of the previous year and choosing those with the higher frequency.

### Data analysis

ED data are collected by the hospital in an administrative electronic database. An intervention variable was added within the monitored data. This variable was automatically selected each time a triage nurse clicked on a DA procedure. In this way we could ensure that the procedure was correctly selected for the selected patients. Data were extracted from the ED electronic database using SAS Software at the end of each month. For each visit, the following variables were recorded: age, gender, symptoms at triage, diagnosis, date and hour of triage registration, priority code, medical imaging, specialist consultations, blood tests, physician’s decisions about the patient, date and hour of hospitalization/discharge. LOS was measured from patient’s registration at the triage to hospitalization/discharge (including boarding time). ED crowding was estimated for each visit through the National ED Overcrowding Study (NEDOCS) score [14]. The NEDOCS score is one tool that is used in Emilia Romagna Region (Italy) and has been found to assess ED overcrowding with relatively high consistency, the NEDOCS was automatically calculated every hour at real-time points [15]. The statistical significance of the differences between intervention and non-intervention weeks was evaluated using Fisher’s exact test for categorical variables, and t-test for continuous variables. Separately for triage conditions, the potential independent association between diagnostic anticipation and ED LOS was evaluated using multiple regression, adjusting for age, gender, priority access codes, specialist consultations, imaging tests, hospitalization and NEDOCS score. All analyses were performed using Stata 15.1 (StataCorp, College Station, Texas, USA, 2017). A two-tailed *p*-value < 0.05 was defined as statistically significant for all analyses.

## Results

**Fig. 1:**
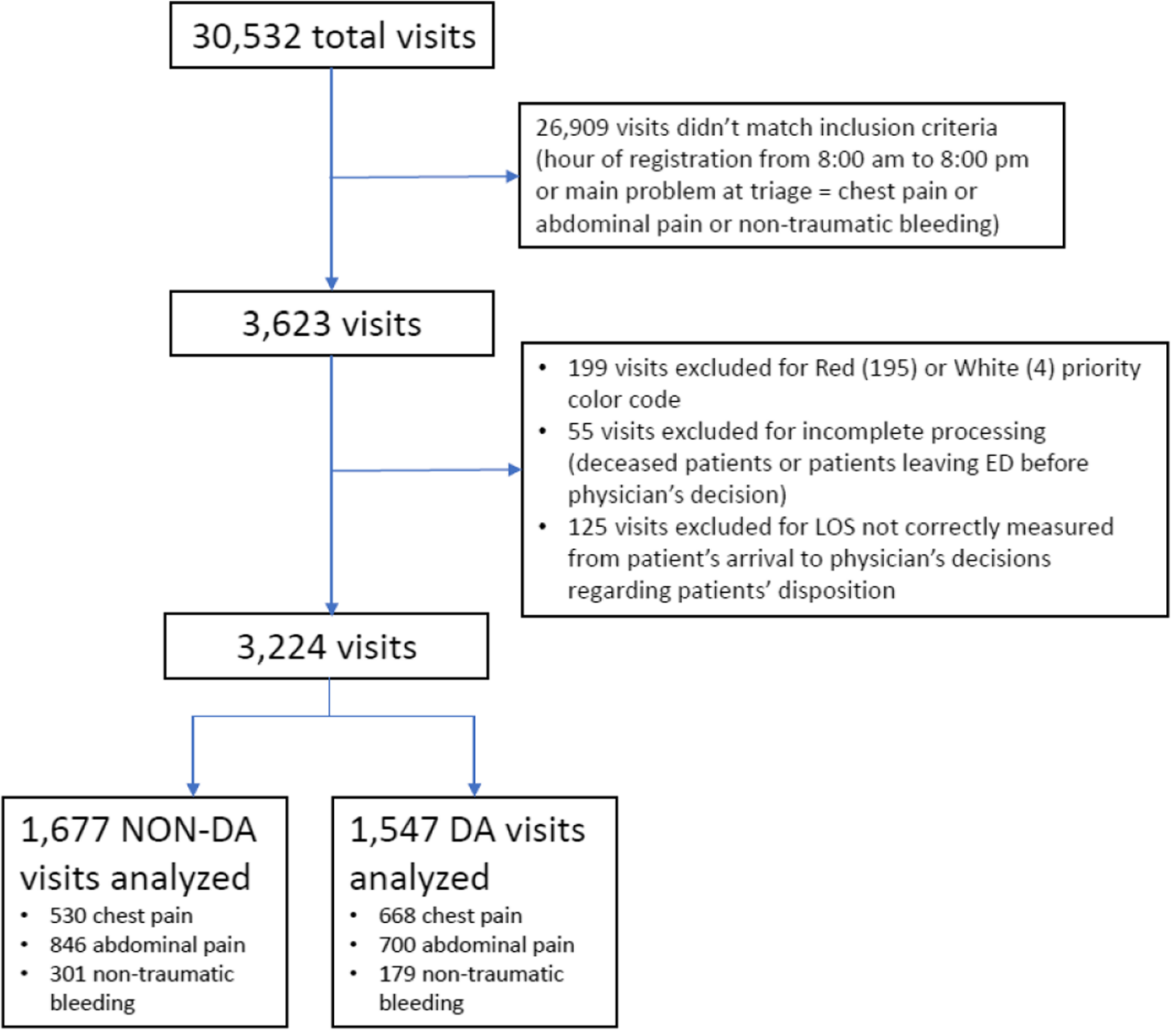
Study profile. Abbreviations: ED = Emergency Department; LOS = Length of Stay; DA = Diagnostic Anticipation

From July 1st 2019 to December 312,019, 3224 visits were included in the study (1677 during control weeks, 1547 during DA weeks), out of a total of 30,532 ED visits (Fig. 1).

As shown in Table 2, some of the demographic and clinical characteristics of the patients significantly differed during DA weeks, as compared with control weeks. In specific, during DA weeks, the NEDOCS score was higher for all clinical conditions, as well as the number of prescribed blood tests (100%), the mean age of the patients with chest pain was slightly lower, whereas patients with non-traumatic bleeding were older by more than 5 years (which probably explains, for these subjects, the higher rate of hospitalization and yellow priority codes).

**Table 2:** Emergency department visits characteristics for presenting complaint at triage registration

Visits characteristics	NON-DA	DA	p-value†
**Chest pain, n**	530	668	
LOS (min), mean (SD)	345 [157]	327 [123]	0.024
Age (years), mean (SD)	60.0 [18.2]	57.5 [19.6]	0.024
Sex (male), %	51.1	48.1	0.29
NEDOCS (score), mean (SD)	127 [59]	157 [67]	< 0.001
Number of imaging tests, mean (SD)	1.9 [0.9]	1.9 [1.0]	0.60
Blood tests (at least 1), %	97.4	100.0	< 0.001
Number of specialist consultations, mean (SD)	0.15 [0.02]	0.14 [0.01]	0.56
Priority color code (yellow), %	84.2	82.8	0.53
Physicians decision (hospitalization), %	18.3	14.1	0.047
**Abdominal Pain, n**	846	700	
LOS (min), mean (SD)	341 [167]	356 [149]	0.053
Age (years), mean (SD)	57.0 [22.3]	56.8 [22.2]	0.88
Sex (male), %	42.3	40.4	0.47
NEDOCS (score), mean (SD)	127 [58]	145 [61]	< 0.001
Number of imaging tests, mean (SD)	1.8 [1.0]	1.9 [0.9]	0.46
Blood tests (at least 1), %	92.6	100.0	< 0.001
Number of specialist consultations, mean (SD)	0.29 [0.02]	0.29 [0.02]	0.75
Priority color code (yellow), %	51.5	56.3	0.06
Physicians decision (hospitalization), %	28.6	27.3	0.57
**Non-traumatic bleeding, n**	301	179	
LOS (min), mean (SD)	304 [147]	337 [166]	0.023
Age (years), mean (SD)	67.9 [20.2]	73.4 [16.5]	0.002
Sex (male), %	61.8	53.6	0.08
NEDOCS (score), mean (SD)	129 [58]	143 [68]	0.016
Number of imaging tests, mean (SD)	1.3 [0.6]	1.4 [0.7]	0.77
Blood tests (at least 1), %	86.4	100.0	< 0.001
Number of specialist consultations, mean (SD)	0.49 [0.03]	0.40 [0.04]	0.09
Priority color code (yellow), %	50.4	62.6	0.004
Physicians decision (hospitalization), %	26.6	35.8	0.034

Abbreviations: *DA* = Diagnostic Anticipation; *LOS* = Length of Stay; *SD* = Standard Deviation; *NEDOCS*=National ED Overcrowding Study Score [15] at triage registration; min = minutes

†calculated through Pearson’s χ2 test for categorical variables and T-Student test for continuous variables

During DA and control weeks, respectively, the following mean ED LOS were recorded (Table 2):

- 327 ± 123 versus 345 ± 157 min for the patients with chest pain (univariate *p* = 0.024);
- 356 ± 149 versus 341 ± 167 min for the patients with abdominal pain (*p* = 0.053);
- 337 ± 166 versus 304 ± 147 min for the patients with non-traumatic bleeding (*p* = 0.023).

Multivariate analyses showed that, for the patients with chest pain, ED LOS was significantly reduced during DA weeks (*p* < 0.001). In contrast, ED LOS did not significantly differ during DA and control weeks for the patients with abdominal pain (*p* = 0.41) and non-traumatic bleeding (*p* = 0.20). The other higher independent predictors of ED LOS were NEDOCS score, number of specialist consultations (at least 1), number of imaging tests, hospitalization (for all patients), age and yellow priority code (only for the patients with chest or abdominal pain), and male gender (for patients with abdominal pain only) (Table 3).

**Table 3:** Multiple regression model predicting ED length of stay

	**Chest Pain**				**Abdominal Pain**				**Non-Traumatic Bleeding**			
	β	95% CI for β		*p*-value	β	95% CI for β		*p*-value	β	95% CI for β		*p*-value
Diagnostic Anticipation	−28.9	−44.2	−13.6	< 0.001	6.1	−8.4	20.7	0.41	17.2	−8.9	43.4	0.20
Age, 5-year increase*	5.7	3.6	7.9	< 0.001	2.4	0.7	4.1	0.006	3.2	−0.2	6.6	0.066
Male gender	−8.1	−22.9	6.8	0.29	−24.1	−38.6	−9.5	0.001	8.3	−17.3	34.1	0.52
NEDOCS score, 10-point increase*	5.0	3.8	6.2	< 0.001	7.0	5.8	8.2	< 0.001	7.3	5.2	9.3	< 0.001
Imaging, 1 test increase*	17.1	9.6	24.6	< 0.001	28.2	21.2	35.2	< 0.001	56.6	41.3	71.8	0.006
Specialist consultations (vs no)	69.2	46.8	91.6	< 0.001	74.7	57.2	92.2	< 0.001	43.4	12.8	73.9	< 0.001
Hospitalization (vs discharge)	52.5	30.4	74.5	< 0.001	126.0	106.8	145.2	< 0.001	94.8	60.5	129.0	< 0.001
Yellow priority code (vs green)	41.2	20.5	61.8	< 0.001	50.6	35.1	66.2	< 0.001	5.8	−21.7	33.3	0.68

Abbreviations: *NT* = Non Traumatic; *β* = β coefficient; *CI* = Confidence Interval, *DA* = Diagnostic Anticipation; *NEDOCS*=National ED Overcrowding Study Score [15] at triage registration

*from the minimum value (0 for NEDOCS and Imaging Tests, 18 for Age)

## Discussion

In this field, retrospective cohort study, the introduction of protocol of DA in ED showed contrasting results: although the ED was reduced by approximately 30 min for the patients presenting with chest pain, no impact was observed for the patients with abdominal pain and non-traumatic bleeding. Also, with regard to chest pain, the observed reduction in LOS was shorter than the mean difference of 51 min reported in a systematic review on triage-nurse ordering [12]. The potential explanations for the observed smaller, or zero impact, are manifold. First, the average ED LOS in the study hospital was long for all patients, approaching 6 h, which may dilute the impact of DA. Second, the studies included in the above mentioned review mostly regarded triage initiated x-rays, and only 2 studies out of 14 also considered blood tests [12]. Moreover, of the two studies including blood tests, one was an unpublished dissertation, and the other had a weak methodology [10]. Third, the DA protocol was implemented for the first time during the 6 months of the study, and the adoption of the algorithm by triage nurses was certainly suboptimal, especially in the first months. Finally, with regard to the different findings on chest pain and abdominal pain or non-traumatic bleeding, this may be due, at least in part, to the lower proportion of blood testing that were performed during control weeks for the subjects with abdominal pain or non-traumatic bleeding, as compared to those with chest pain. Performing a lower number of blood tests could clearly result into a shorter LOS, jeopardizing the potentially positive impact of anticipation. Certainly, further research is needed to clarify these points, as well as to confirm of disprove the benefit of DA for the patients with chest pain.

The other results of the multivariate analyses were straightforward: a longer ED LOS was observed for patients, with upper priority code, during the periods of higher ED crowding (higher NEDOCS score). Noteworthy, female patients with abdominal pain showed a significantly longer LOS than males. This could be explained by the fact that abdominal pain has gender-specific diagnostic differences (for example gynecological conditions). Again, further, specific studies are warranted to investigate the potential gender difference on LOS and its potential organizational consequences.

### Limitations

First, in this study the DA protocol was limited to the daily hours of service from 8:00 am to 8:00 pm, due to a limited availability of resources (nurses in service) during night shifts. However, during the nights, ED crowding is typically lower.

Second, triage-initiated blood testing requires a crowded ED in order to detect a positive impact on LOS: in uncrowded ED patients are immediately, or after a very short waiting time, addressed to physician’s evaluation, and it may not be observed any LOS reduction from anticipated testing. In this study, the mean NEDOCS score ranged from 120 (overcrowded) to 160 (severely overcrowded). Thus, the findings of this study cannot be generalized to ED with low crowding status and short waiting times before physician’s evaluation.

## Conclusions

The introduction of a protocol of DA of blood tests at triage, into a crowded ED, showed contrasting results: the LOS was significantly reduced, by approximately 30 min, for the patients reporting chest pain, whereas no impact was observed for the patients with abdominal pain or non-traumatic bleeding. Although the impact of diagnostic anticipation could be substantial in reducing the ED waiting time, further research is required to confirm the positive findings and investigate the potential reasons of the observed discrepancies by clinical condition.

## Supplementary information

**Additional file 1.**

## Data Availability

The datasets used and analyzed during the current study are available from the corresponding author on reasonable request.

## Abbreviations

ED: Emergency Department
LOS: Length Of Stay
ALT: Alanine Transaminases
CRP: C-Reactive Protein
PT: Prothrombin Time
PTT: Partial Thromboplastin Time
NEDOCS: National Emergency Department OVercrowding Study
DA: Diagnostic Anticipation
SD: Standard Deviation
NT: Non Traumatic
β: β coefficient
CI: Confidence Interval
