# Supplementary material for: Diagnostic anticipation to reduce emergency department length of stay: a retrospective cohort study in Ferrara University hospital, Italy
Source: BMC Health Serv Res. 2020 Jul 8;20:624. doi: 10.1186/s12913-020-05472-3 (PMC7346651; doi:10.1186/s12913-020-05472-3)
Supplement: Supplementary file 1 — Additional file 1. [file 12913_2020_5472_MOESM1_ESM.pdf]

Additional files: case definition for presenting complaints

CHEST PAIN

- SHOCK
- PAS < 90 MMHG, FC < 40/MIN O > 150/MIN
- FR < 10/MIN O > 34/MIN E/O SPO2 < 90%
- ST elevation MI in ECG (STEMI)

→ **RED**

NO ↓

- CHEST PAIN WITHOUT SHOCK
- CHEST PAIN WITHIN THE LAST 6 HOURS
- CHEST PAIN IN CHRONIC ISCHEMIC HEART DISEASE
- CARDIOVASCULAR RISK FACTORS
- TVP/TEP RISK FACTOR
- COCAINE USE

→ **YELLOW**

NO ↓

- ATYPICAL PAIN WITHOUT CARDIOVASCULAR RISK FACTORS
- ATYPICAL PAIN LASTING MORE THAN 6 HOURS

→ **GREEN**

ABDOMINAL PAIN

- SHOCK
- PAS < 90 MMHG
- FC < 40/MIN O > 150/MIN
- ABDOMINAL ANEURYSM KNOWN
- PULSATING ABDOMINAL MASS
- ABDOMINAL STIFFNESS AT PALPATION

→ **RED**

NO ↓

- NORMAL ABDOMEN (PAIN SCORE ≥7)
- DIARRHEA
- VOMITING
- TEMP ≥ 38° C
- RECENT ABDOMINAL SURGERY

→ **YELLOW**

NO ↓

- NORMAL ABDOMEN
- PAIN SCORE ≤ 6

→ **GREEN**

NON TRAUMATIC BLEEDING

- ACTIVE HEMATEMESIS
- SHOCK
- PAS < 90 MMHG
- FC > 130/MIN

→ RED

NO ↓

- ACTIVE MELENA OR RECTORRHAGIA
- HEMATEMESIS WITHIN THE LAST 24 HOURS
- AMDOMINAL PAIN
- HEMATEMESIS, MELENA OR RECTORRHAGIA (>24 HOURS) WITH RISK FACTORS: ORAL ANTICOAGULANT THERAPY, COAGULOPATHY, CIRRHOSIS, CANCER

→ YELLOW

NO ↓

- HISTORY OF GI BLEEDING
- LOWER GI BLEEDING (MINOR)

→ GREEN
